# Childhood Immunisation Coverage during the COVID-19 Epidemic in Italy

**DOI:** 10.3390/vaccines10010120

**Published:** 2022-01-14

**Authors:** Michela Sabbatucci, Anna Odone, Carlo Signorelli, Andrea Siddu, Andrea Silenzi, Francesco Paolo Maraglino, Giovanni Rezza

**Affiliations:** 1 Department Clinical and Experimental Medicine, University of Pisa, 56126 Pisa, Italy; 2 Department Infectious Diseases, National Institute of Health, 00161 Rome, Italy; 3 Ministry of Health, Directorate General Health Prevention, Communicable Diseases and International Prophylaxis, 00144 Rome, Italy; a.siddu@sanita.it (A.S.); a.silenzi@sanita.it (A.S.); f.maraglino@sanita.it (F.P.M.); g.rezza@sanita.it (G.R.); 4 Department of Public Health, Experimental and Forensic Medicine, University of Pavia, 27100 Pavia, Italy; anna.odone@unipv.it; 5 School of Medicine, Vita-Salute San Raffaele University, 20132 Milan, Italy; signorelli.carlo@hsr.it

**Keywords:** vaccine coverage rates, Italy, paediatric immunisation, COVID-19 impact, childhood vaccine uptake

## Abstract

The COVID-19 pandemic has affected national healthcare systems worldwide, with around 282 million cumulative confirmed cases reported in over 220 countries and territories as of the end of 2021. The Italian National Health System was heavily affected, with detrimental impacts on preventive service delivery. Routine vaccination services were disrupted across the country during the first months of the pandemic, and both access to and demand for vaccines have decreased during the pandemic. In many cases, parents preferred to postpone scheduled appointments for routine paediatric vaccinations because of stay-at-home orders or fear of COVID-19 infection when accessing care. The objective of the current study was to assess the routine childhood vaccine coverage (VC) rates during the COVID-19 epidemic in Italy. We compared 2020 and 2019 VC by age group and vaccine type. The Italian Ministry of Health collected anonymised and aggregated immunisation national data through the local health authorities (LHAs). Results were considered statistically significant at a two-tailed *p*-value ≤ 0.05. VC rates for mandatory vaccinations decreased in 2020 compared to 2019 (range of VC rate decrease: −1% to −2.7%), while chicken pox increased (+2.2%) in 7-year-old children. Recommended vaccinations were moderately affected (range of VC rate decrease in 2020 vs. 2019: −1.4% to −8.5%), with the exception of anti-HPV in males, Men ACWY, and anti-rotavirus vaccination (VC increase 2020 vs. 2019: +1.8%, +4.7% and +9.4%, respectively). In the COVID-19 era, the implementation of coherent, transparent, and effective communication campaigns and educational programs on safe childhood vaccinations, together with the increase in the number of healthcare staff employed, is essential to support strategies to reinforce vaccination confidence and behaviour, thus avoiding health threats due to VPD during and beyond COVID-19 times.

## 1. Introduction

The SARS-CoV-2 infection has spread widely at the global level in 2020, causing disruption of routine vaccination activities in most countries [1,2]. International health authorities revealed a decreased demand for vaccination because of physical and social distancing requirements or community reluctance due to the fear of contagion. The World Health Organisation (WHO) published interim guidelines [3,4] warning about the risk of vaccine-preventable disease (VPD) outbreaks, which could cause further pressure on health services. The COVID-19 pandemic had a critical impact on Italy’s population and national health system. After the first case detected in Lombardy Region on 21 February 2020, SARS-CoV-2 infection continued to spread throughout the country. The number of new cases peaked on 21 March 2020 and then decreased progressively due to strict nonpharmacological preventive measures implemented locally and at the national level [5]. Public health interventions to reduce the spread of SARS-CoV-2 infection focused on the stay-at-home policy and social distancing, with general lockdown or restricted mobility, closure of nonessential services including schools, and implementation of protocols such as mask-wearing, as well as regular and correct hand hygiene. Health and social workers were forced to face professional procedures and management strategies at the organisational and structural level, experiencing bio-risk activities, shortage of disposable personal protective equipment, the limited possibility of replacement and rostering, personal discomfort, prolonged stress, and burnout. Cancer patients have been experiencing treatment rescheduling, while elective surgical care has been facing disruption of appointments and consequent delays, resulting in fear and stress [6,7]. Some healthcare resources were shifted to the COVID-19 response, including specific staff training and the administration of COVID-19 vaccines, with a considerable impact on routine immunisation activities. During both the acute and the post-acute phases of the health emergency, the decreased offer of and demand for routine vaccines [8,9] and the reduced availability of health workers heavily affected routine immunisation services. The Italian vaccination policy applies across the life-course, with the following 10 mandatory routine immunisations for children aged 0–16 years and unaccompanied foreign children [10]: polio, diphtheria, tetanus, hepatitis B, pertussis, *Haemophilus influenzae* type b, measles, rubella, mumps, and chicken pox [11]. Furthermore, the Italian Ministry of Health recommends vaccinations against human papillomavirus (HPV) and meningococcus (serotypes A, B, C, Y, W135) for adolescents, as well as herpes zoster (HZ) and pneumococcus for the population over 65 years. The National Vaccine Prevention Plan (PNPV) 2017–2019 (extended to 2021), together with the Essential Level of Assistance (LEA), provides free HPV vaccination during the 12th year of age for both females and males. Mandatory and recommended vaccinations are offered free of charge according to the age ranges established in the national vaccine calendar. Here, we assessed the national vaccine coverage (VC) rates reached in 2019 with those achieved during the first epidemic year to investigate the extent of the slowdown in routine paediatric vaccination activities in 2020 and to identify which infectious diseases could give rise to outbreaks in the near future eventually.

## 2. Materials and Methods

The Italian Ministry of Health collects yearly anonymised and aggregated data on administered vaccines (numerator) and the target resident population (denominator) of the same age (i.e., per birth cohort) through the local health authorities (LHAs) from the 19 Regions and the two Autonomous Provinces (R/AP) according to their organisation models. Data are collected at 24 months and 7 years of age each year. Since 2019, the Ministry of Health has also collected data for rotavirus vaccination at 12 months of age.

In this paper, data on national coverage rates were analysed using the same methodologies used in the past to compare 2019 and 2020 data. We used polio and measles as the usual proxy for the hexavalent and trivalent vaccinations, respectively, since these vaccines are administered in six-in-one and three-in-one vaccine formulations in Italy. Data are reported per birth cohort and a complete vaccination cycle (regardless of the schedule adopted and the vaccine type administered). As for polio and pneumococcus (PNC) vaccination, we used the available data at 24 months of age, even though these vaccines are administered at 3, 5, and 11 months of age, comparing VC rates for the years 2019 (administered from January to December 2019 to the 2017cohort) and 2020 (to the 2018cohort). We used data on measles, chicken pox, and meningococcus (Men B, Men C, Men ACWY) vaccinations in 2020 (VC 2020) to the 2018 cohort (24 months old) and the 2013 cohort (7 years old), as well as data on vaccinations administered through the year 2019 (VC 2019) to the 2017 and 2012 cohorts, as these vaccines are administered at around 13–15 months (for measles, chicken pox, and Men C), at 3, 5, 7, and 13 months of age (for Men B), or at 12 and 18 months of age (for Men ACWY). Polio, measles, and chicken pox vaccines are boosted at 6 and at around 16 years of age (between 11 and 18 years of age), mandatorily. Here, we evaluated the first two timepoints (24 months and 7 years of age) only, as the fourth dose has a massive range of possible administration that would not indicate the impact of the COVID-19 epidemic on the VC rates achieved in 2020.

Vaccination against rotavirus is administered in Italy within the first year of age. Therefore, we compared VC rates at the 12-month timepoint (VC 2019 for the 2018 cohort and VC 2020 for the 2019 cohort).

Regarding anti-HPV vaccination, we used the full-cycle coverage for 13-year-old males and females (2006 cohort in 2019, 2007 cohort in 2020) since it is offered free of charge between the 11th and 12th birthdays.

We performed a statistical analysis at 12 or 24 months and 7 years for mandatory and recommended vaccinations. The chi-square test was executed on proportions for the years 2020 vs. 2019 using the SPSS statistical program, version 12.0 for Windows (IBM, Armonk, NY, USA). Analysis findings were considered statistically significant at a two-tailed *p*-value ≤0.05.

## 3. Results

The 2019–2020 national-level immunisation coverage rates for mandatory vaccinations are reported in Table 1. We reported the number of vaccinated children (numerator) and the eligible paediatric population (denominator) registered in Italy for each VPD and year of administration.

In 2020, 24-month coverage rates were 94.0% for polio, 92.7% for measles, and 90.3% for chicken pox, representing −1.0%, −1.8%, and −0.2% decreases, respectively, as compared to 2019. By 7 years of age, immunisation coverage was 85.9% for polio, 85.8%, for measles, and 40.6% for chicken pox, representing a −2.7%, −1.8%, and +2.2% difference, respectively, compared to 2019.

With reference to the recommended childhood immunisations, in 2020, 24-month coverage was 66.3% for Men B (−2.7% as compared to 2019), 71.0% for Men C (−8.5%, as compared to 2019), 51.3% for Men ACWY (+4.8% as compared to 2019), 90.6% for pneumococcal vaccine (−1.4%, as compared to 2019), and 62.8% for rotavirus (+36.7%, as compared to 2019). With regard to HPV vaccination, in 2020, we registered a decrease in VC for adolescent females (−2.2%), but an increase for males (+1.8%) (Table 2).

Overall, the VC threshold goal was reached neither in 2020 nor in the previous year, apart from polio (hexavalent) and almost measles (trivalent) in 2019. The national reduction in VC rates observed during the COVID-19 epidemic was minor even if statistically significant for the mandatory vaccinations (ranging from −1% to −2.7% decrease) and for some of the recommended vaccinations (−1.4% for PNC, −2.2% for HPV in females, and −2.7% for Men B vaccinations), and it might be regained in the near future.

Critically, Men C (−8.5%) registered a significant and severe VC reduction in 2020 compared to 2019.

Interestingly, we observed a significant positive increase for HPV in males, Men ACWY, and rotavirus vaccinations (+1.8%, +4.7%, and +9.4%, respectively) in 2020 compared to 2019.

## 4. Discussion

Immunisation is a core component of public healthcare assistance and citizens’ safety. However, during the current COVID-19 pandemic, many countries have struggled to maintain routine essential services.

In Italy, the COVID-19 emergency has diverted health resources from preventive care. It has disrupted not only the activities in acute and emergency healthcare facilities but also the overall services offered by the national health system and by the regional health services, including routine childhood immunisation activities [9,12,13].

Our data show that VC rates for mandatory vaccinations slightly decreased in 2020 compared with the previous year (range −1.0% to −2.7%), with the exception of chicken pox vaccination that showed an increase (+2.2% in 2020 vs. 2019). Most recommended vaccinations underwent a moderate decrease (range −1.4% to −2.7%). However, anti-Men C suffered the epidemic impact the most (−8.5%), while VC rates for anti-HPV in males, anti-Men ACWY, and anti-rotavirus improved (+1.8%, +4.8%, and +9.4%, respectively). Therefore, in Italy, five mandatory and four recommended paediatric vaccinations slowed down during the epidemic waves in 2020, while the administration of one mandatory and three recommended vaccinations increased significantly compared to the previous year. Chicken pox vaccination became mandatory with the 2017 cohort onwards; this might explain the favourable trend.

Some strategies to mitigate barriers to immunisation during the epidemic have been implemented in Italy, i.e., environmental sanitation and room ventilation, access to vaccination services by telephone appointment to avoid crowds, opening hours at different time slots to increase availability, optimisation of spaces dedicated to vaccine administration and active search for new locations, other than detection of body temperature and any respiratory symptom, and advice on hand hygiene in individuals accessing vaccination services, together with local information on SARS-CoV-2 infection. Additional strategies should be implemented, involving primary and community health providers [14], universal centralised electronic immunisation records [15], immunisation reminders for needed vaccinations [16,17], full transparency on vaccine safety, and educational campaigns for parents, which could increase vaccine uptake and VC rates, thereby reducing the risk of outbreaks due to VPD. Moreover, assuring appropriate planning of medical and nonmedical postgraduate residency programs in the health sector, as well as implementing the number of health workers employed to support health service activities overall and routine immunisation activities specifically, would enforce the preparedness of the entire healthcare system to face health threats. Moreover, activation of periodic or regular active surveillance for all childhood vaccinations would reinforce population trust in vaccination behaviour and facilitate adherence to national public health immunisation programs. Furthermore, offering effective communication and coherent health messages for the general public as well as promoting educational courses on vaccinology targeted to all the health professionals would build and maintain the confidence needed in the capacity of the health system to safely meet essential needs and to control the infectious risk, while guarantying equitable access to vaccinations to hard-to-reach populations, including migrants.

Our study had some limitations. We evaluated VC rates for polio at 24 months of age (cohort 2018 in 2020), as the Italian Ministry of Health collects data yearly at 24 months and 7 years of age. However, children born in 2018 received their polio vaccination in 2019; therefore, in 2020 we evaluated the late administrations in addition to the due optimal appointments. This allowed us to assess the VC rates for polio vaccination including the delayed administrations. Furthermore, every year, VC rates vary considerably between R/AP. Some of them (particularly some regions in northern Italy, i.e., Lombardy, Veneto, Emilia-Romagna, and Piedmont) were affected early and more severely by the COVID-19 epidemic. Assessing the VC rates by year and nationally allowed overall evaluation of the paediatric VC state in Italy. We can also consider that some of the areas most affected by the COVID-19 epidemic are those most compliant traditionally with vaccination needs; therefore, these regions are thought to be able to promptly administer the doses missed during these epidemic years.

While immunisation programs are struggling to achieve optimal coverage targets, during 2020, prolonged lockdown, limited access to healthcare services, and fear of SARS-CoV-2 possible contagion, along with vaccine hesitancy [18,19], impacted childhood immunisation uptake [20]. Despite a moderate decrease in VC rates, we registered no increase in outbreaks due to VPD. Until the COVID-19 pandemic, Italy was one of the countries in the European Region where a large measles outbreak occurred despite the targets outlined in the National Plan to eliminate measles and congenital rubella [21]. However, 103 cases of measles were reported in Italy (incidence 3.4 cases per million, median age 33 years) between January and March 2020, with no reported measles cases from April to December 2020, together with 16 cases of rubella (median age 30 years) reported between January to October 2020 [22]. In 2021, from 1 January to 31 August, no cases of rubella and three cases of measles (one laboratory-confirmed and two classified as possible cases) were reported in Italy (incidence: 0.1 cases per million population) [23]. On the contrary, 1627 cases of measles (incidence: 27 cases per million, median age 30 years) and 22 cases of rubella (median age 28 years) were reported in 2019. Therefore, even if VC rates for measles decreased by about 2% at both 24-month and 7-year timepoints in 2020 vs. 2019, we did not register an increase in the number of measles cases in 2020 or in 2021. Similarly, we observed a marked decrease ranging from around −60% to −80% in the number of cases for chicken pox, meningococcal disease, and pneumococcal infections in 2020 as compared to the previous year, despite the specific VC rates decreasing in 2020 respect to 2019. While the COVID-19 epidemic has hindered or slowed routine vaccinations, the impact on public health has so far been more than balanced by the use of personal protective equipment, frequent hand hygiene, quarantine/isolation periods, and physical distancing, which likely prevented the spread of other respiratory diseases as well.

Other countries reported a slight decrease in VC rates against paediatric infectious diseases [24] and a reduced overall incidence of VPD [25,26,27], while some countries experienced a marked decrease in VC rates [28,29,30,31,32,33], slow resumption of immunisation services [34,35,36,37], or an overall negative pandemic impact on their immunisation services [38]. Indeed, the pandemic has led to global disruptions in essential health services. Amidst the fast-expanding COVID-19 literature, there is little comprehensive coverage of the pandemic’s indirect impact on child health. Although the latest VC trajectories point towards recovery in some regions, a combination of lagging catch-up immunisation services, continued SARS-CoV-2 transmission, and previous gaps in VC before the pandemic still left millions of children under-vaccinated or unvaccinated against VPD at the end of 2020 [1,2,39,40], with the most severe burden in North Africa, the Middle East, South Asia, Latin America, and the Caribbean. Strengthening efforts to target resources and routine immunisation activities will be essential to minimise the risk of VPD outbreaks in every country. In this regard, the WHO [3,4] recommends maintaining vaccination activities and prioritising primary series vaccinations, especially for measles, rubella, and poliomyelitis, and the immunisation services, and to evaluate current national epidemiology of VPD and the COVID-19 local transmission scenario. Maintaining high VC is even more important during the COVID-19 crisis to avoid the resurgence of VPD [41] and mitigate the risk of health system collapse, especially where the magnitude and impact of under-immunisation are severe.

## Figures and Tables

**Table 1:** National vaccine coverage (VC) rates (%) registered for mandatory vaccinations, plus chicken pox at 24 months and 7 years of age, stratified by vaccine type and year of administration, along with the percentage differences between the 2019 and 2020 rates in Italy.

Target Age Group	VPD	VC Rates (%) by Year of Vaccine Administration		% Difference (2020 vs. 2019)	*p*-Value *
		2019	2020		
24 months	Polio	95.01 (431,723/454,396)	94.02 (408,620/434,596)	−0.99	<0.001
	Measles	94.49 (429,338/454,361)	92.70 (402,868/434,596)	−1.79	<0.001
	Chicken pox	90.50 (411,206/454,361)	90.28 (392,329/434,588)	−0.22	<0.001
7 years	Polio	88.62 (473,491/534,277)	85.92 (438,304/510,132)	−2.70	<0.001
	Measles	87.58 (467,940/534,277)	85.82 (439,738/510,132)	−1.76	<0.001
	Chicken pox	38.36 (204,974/534,277)	40.56 (208,843/510,132)	+2.20	<0.001

* Chi-square test (2020 vs. 2019); VC: vaccination coverage; VPD: vaccine-preventable disease; data source: data from the Italian Regions/Autonomous Provinces.

**Table 2:** National vaccine coverage (VC) rates (%) registered for recommended vaccinations at 12 months, 24 months, and 13 years of age, stratified by vaccine type and year of administration, along with the percentage differences between the 2019 and 2020 rates in Italy.

Target Age Group	VPD	VC Rates (%) by Year of Vaccine Administration		% Difference (2020 vs. 2019)	*p*-Value *
		2019	2020		
12 months	Rotavirus	60.94 (265,168/435,162)	70.34 (291,017/413,728)	+9.40	<0.001
24 months	Men B	68.98 (313,721/454,775)	66.30 (288,154/434,596)	−2.68	<0.001
	Men C	79.44 (361,288/454,775)	70.96 (308,371/434,596)	−8.48	<0.001
	Men ACWY	46.58 (211,836/454,775)	51.27 (222,797/434,596)	+4.69	<0.001
	PNC	92.00 (418,380/454,775)	90.58 (393,655/434,596)	−1.42	<0.001
13 years	HPV (females)	60.83 (168,680/277,302)	58.66 (160,219/273,154)	−2.17	<0.001
	HPV (males)	44.84 (130,907/291,966)	46.61 (135,249/290,170)	+1.77	<0.001

* Chi-square test (2020 vs. 2019); HPV: human papillomavirus; Men B, C, and ACWY: *N. meningitidis* serogroups B, C, and ACW135Y; PNC: *S. pneumoniae*; VC: vaccination coverage; VPD: vaccine-preventable disease; data source: data from the Italian Regions/Autonomous Provinces.

## Data Availability

Data supporting reported results can be found at http://www.salute.gov.it/portale/documentazione/p6_2_8_3_1.jsp?lingua=italiano&id=20 (accessed on 4 December 2021).
